# Electroacupuncture Is Effective for Peripheral Facial Paralysis: A Meta-Analysis

**DOI:** 10.1155/2020/5419407

**Published:** 2020-03-31

**Authors:** Wei-Hua Wang, Ruo-Wen Jiang, Na-Chuan Liu

**Affiliations:** ^1^Second Affiliated Hospital of Heilongjiang University of Chinese Medicine, Harbin, Heilongjiang 150001, China; ^2^Heilongjiang University of Chinese Medicine, Harbin, Heilongjiang 150040, China

## Abstract

**Objective:**

To explore the status of electroacupuncture (EA) among other treatments for peripheral facial paralysis (PFP).

**Methods:**

Randomized controlled trials comparing EA with other treatments that met the eligibility criteria published in databases were included. The differences were observed and quantified through the risk ratio (RR) for dichotomous outcomes and the standardized mean difference (SMD) for continuous outcomes. Then, their 95% confidence intervals (CI) were recorded.

**Results:**

Twenty-three studies involving 1985 participants were included. META-analysis results showed that EA was better than manual acupuncture for PFP (RR: 1.16, 95% CI 1.11 to 1.22, for responding rate; SMD: 2.26, 95% CI 0.15 to 4.37, for facial nerve function) and current promoted recovery (RR: 1.21, 95% CI 1.15 to 1.27, for responding rate; SMD: 2.87, 95% CI 1.16 to 4.58, for facial nerve function). When combined with other treatments, EA improved their effectiveness (RR: 1.19, 95% CI 1.12 to 1.28, responding rate; SMD: 1.85, 95% CI 0.67 to 3.03, facial nerve function).

**Conclusion:**

Patients with PFP received EA (used separately or combined with other treatments) resulting in a better prognosis. However, the quality of evidence was very low-to-moderate. Considering the poor quality of evidence, we are not very confident in the results. We look forward to more research and update results in the future and improve the evidence quality.

## 1. Introduction

Peripheral facial paralysis (PFP) is a class of facial paralysis characterized by the dyskinesia of facial muscles by which affected patients may develop facial asymmetry persisting for weeks to months. Additional symptoms of PFP include ear pain and facial numbness. The incidence is described as 11.5 to 40.2 per 100000 people a year [1–3]. However, the exact etiology and pathogenesis of PFP are still unknown. Currently, it is thought that the herpes viruses (herpes simplex virus, herpes zoster virus, or both) may play a key role [1, 2]. Another possible contributor to the pathogenesis implicates the role of a cell-mediated immune response against myelin, akin to a mononeuropathy form of Guillain–Barré syndrome (GBS) [2].

There is a lot of strong evidence recommending the use of corticosteroids for PFP, as it can improve short-term and long-term prognosis [3–6]. Considering the key role that herpesviruses may play, the antivirals are also used although there is no strong evidence to prove its benefits [5–7]. Many researchers are trying more treatments to bring better prognosis to patients, such as exercise, physiotherapy, electrostimulation, surgical decompression, and eye-protective measures for incomplete eye closure [6].

Acupuncture, as a Traditional Chinese Medicine (TCM) therapy that has persisted throughout history, can effectively treat facial paralysis based on some studies. Acupuncture can improve House–Brackmann (HB) and Sunnybrook (SB) scores in patients with Bell's palsy [8]. There are multiple acupuncture methods that exist for this purpose (including electroacupuncture (EA), manual acupuncture (MA), warm needling (or moxibustion-acupuncture), and stuck needling) [9]. EA seems to be a treatment that combines MA and electrostimulation. In China, many studies have claimed that the curative effect of EA on PFP is significant [10, 11] and EA is also suitable for the treatment of sequelae of PFP [12]. However, there is no enough evidence to support these claims.

We hope to explore the status of EA therapy in PFP treatment and the benefits or harm it may bring to PFP patients. To resolve the dispute over the benefits or harm of EA in patients with PFP, the goal of this meta-analysis is to explore for evidence and assess the effects of EA for PFP. We primarily focused on the differences in the responding rate (primary outcome) between EA and other treatments. In addition, FNF was regarded as a secondary outcome, measured by HB and SB scores and electromyography (EMG). Adverse events were also recorded.

## 2. Methods

### 2.1. Eligibility Criteria

As a clinical diagnosis, the characteristics of PFP are acute onset of unilateral lower motor neuron facial paralysis that affects muscles of the upper as well as the lower face and reaches its peak by 72 hours. Symptoms are frequently accompanied by neck, oropharyngeal, or facial numbness, mastoid or ear pain, hyperacusis or altered facial sensation, and disturbed taste on the anterior part of the tongue [1, 3].

Studies which met all of the following requirements were included: 1. randomized controlled trials (RTCs) comparing EA with other treatments; 2. participants were diagnosed with PFP by clinical doctors according to the diagnostic criteria in the original study; 3. the experimental group (EA group) received EA whether or not combined with treatments received in the control group; and 4. for multiple reports of the same research, we only included the latest report.

Studies were excluded if 1. the data was incomplete or inconsistent; thus, no valid data for primary outcome could be extracted; 2. the paper was retracted; 3. interventions used in the EA group (except EA) were not used in the control group; and 4. the diagnostic criteria of the original study did not meet with the clinical diagnosis of PFP.

### 2.2. Literature Searching, Screening, Evaluation of Bias, and Data Extraction

We searched the following databases: China National Knowledge Infrastructure (CNKI), Wanfang Databases, VIP databases, Cochrane Library, ScienceDirect, and PUBMED using the keywords (in Chinese for Chinese databases) as follows: [(Bell's palsy OR facial paralysis OR facial palsy OR facial nerve paralysis) AND electroacupuncture AND trial]. We also searched for any clinical trial registrations at the International Clinical Trials Registry Platform (ICTRP).

An author scrutinized the search databases to identify studies. Two authors independently reviewed the full text to determine whether the studies met the criteria. We assessed methodological bias with the Cochrane risk assessment and evaluated publication bias through the funnel chart. The quality evaluation and data extraction were completed by all three authors independently. Inconsistences were resolved through group discussion.

The extracted data included information such as the sample size (number of participants), age (years), gender, course of the disease (days), number of arms, responding rate, adverse events, posttreatment scale scores, and examination results. The primary outcome was the responding rate of EA and other alternative treatments, and the secondary outcomes were the FNF and adverse events. To determine whether a treatment was effective, the standard of effective we used referred to the objective assessment standards used by the original study (for example, 1 level improvement in HB grading scale). In the meantime, we evaluated FNF through quantitative data (HB, SB, EMG, etc.).

According to the GRADE approach [13], we divided the evidence into four levels: high, moderate, low, and very low. High quality means that we were very confident about the results; very low quality means that we believed that the results would be easily overturned by future researches.

### 2.3. Statistical Analysis [14]

We analyzed data with Office Excel 365 and Review Manager 5.3 (RevMan 5.3). We used mean ± standard deviation to describe measurement data (age, course of the disease, etc.). We measured the responding rate with the risk ratio (RR) and its 95% confidence interval (95% CI). Considering the different scales for FNF used in original studies, we calculated the standardized mean difference (SMD) and its 95% CI to observe the continuous outcome (scale scores). We used the following method to uniform the measurement direction of the FNF (the higher the score and the better the FNF): if the original direction was inconsistent with the target direction, multiply the mean of the score by −1 and keep the standard deviation.

We assessed the heterogeneity in clinical and methodological aspects through discussion. Also, we performed the *I*^2^ test to assess homogeneity of outcomes. An outcome was considered homogeneous if *I*^2^ < 40%. Otherwise, heterogeneity was further explored using sensitivity analysis or subgroup analysis.

## 3. Result

### 3.1. Study Selection and Study Characteristics

**Figure 1:**
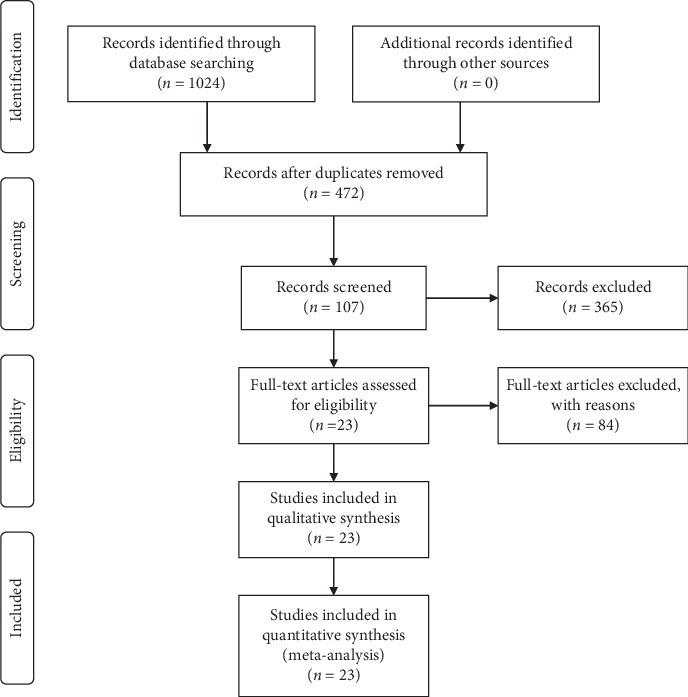
Flow diagram of the literature selection process.

We performed the search in January 2020. In a total of 1024 returned records, we did not find unreported trials that met the eligibility criteria in ICTRP. After removing duplicate records, we identified 107 studies from 472 studies for review of the full text. Finally, 23 were included in this meta-analysis (Figure 1).

The final 23 studies involved 1985 participants (male/female: 994/991) in 11 provinces in China (Table 1). Overall, the participants' age range was 1 month to 70 years (37.92 ± 17.78 years). One study included children (less than 18 years of age), 10 studies included adults (18 years of age and older), and 8 studies included subjects of all ages. Four studies did not report the age range of the participants. Seven studies included patients in the acute phase within 14 days after the onset, three studies included patients with a course of more than 30 days, and 4 studies did not report the range of the courses.

These studies covered a wide range of age and course of the disease. Gender was balanced throughout the studies.

In each study, acupoints were the similar across groups. The main acupoints were on the affected side. The most widely used acupoint was Yang-bai (GB14, 22/23), followed by Di-cang (ST4, 21/23), Jia-che (ST6 20/23), Yi-feng (SJ17, 16/23), Ying-xiang (LI20, 16/23), Quan-liao (SI18, 15/23), Xia-guan (ST7, 14/23), Cuan-zhu (BL2, 13/23), Tai-yang (EX-HN5, 12/23), Si-bai (ST2, 12/23), Cheng-jiang (RN24, 10/23), Yu-yao (EX-HN4, 10/23), etc.

### 3.2. Study Design and Risk of Bias

All 23 studies were RTCs, and the sample sizes were small to medium (Table 2). The control group received “controls” other than EA, including manual acupuncture and standard drugs. In addition, the experimental group received EA, combined with “controls” or used separately.

**Figure 2:**
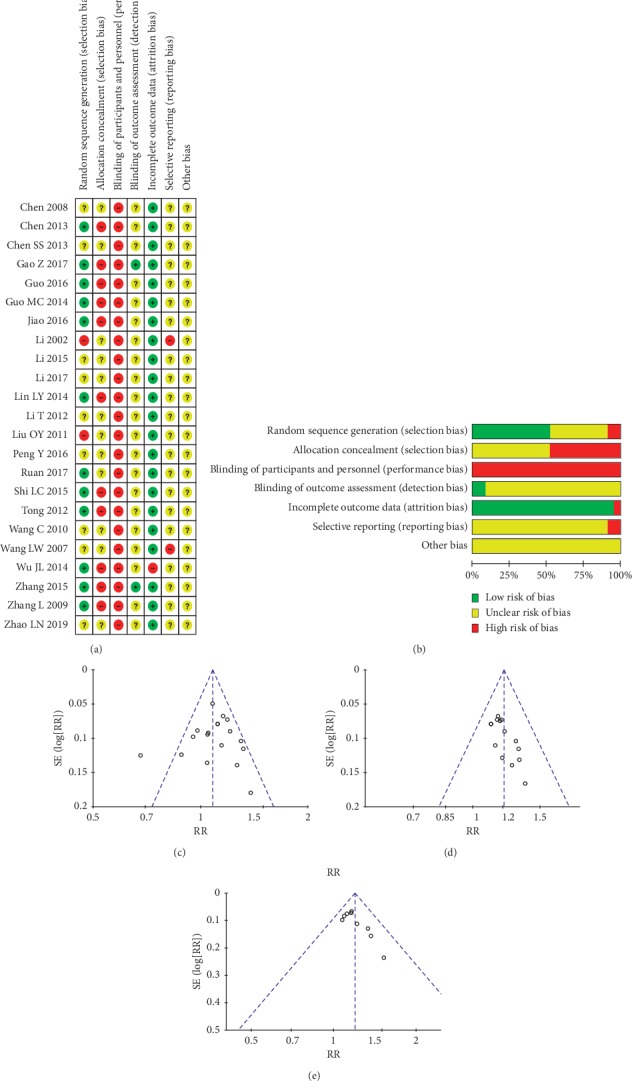
(a) Risk of bias summary. (b) Risk of bias graph. (c) Funnel chart of EA versus “controls”. (d) Funnel chart of current. (e) Funnel chart of EA as an adjunct.

All studies included had various methodological defects (Figures 2(a) and 2(b)), mainly the incompleteness in randomization and blinding. Besides, there were problems such as imperfect trial registration and possible selective reporting. Eleven studies [16, 18–21, 26, 30, 31, 34–36] used the random number table to generate random numbers; 2 studies [22, 27] generated random numbers with high risk of bias (using the order of visit or the order of selection); 1 study [29] used random numbers generated by using a computer, and the other studies [15, 17, 23–25, 28, 32, 33, 37] did not clarify the method of random number generation. All studies' allocation concealment was either not effective or not reported. None of the included studies performed “sham acupuncture” for comparison. Only 2 studies [18, 35] used the blinding at the measurement stage to reduce detection bias. Two studies [22, 33] were assessed at a high risk of bias in selective reporting since some outcomes reported were not stated in advance (methods section).

After discussion, we decided that heterogeneity in characteristics and methodologies were not high enough to prohibit the results be pooled together. We tested the stability of the outcomes by sensitivity. The funnel chart of EA versus “controls” was roughly symmetrical (Figure 2(c)). We decided that publication bias would not affect the quality of the effects of EA versus “controls” through discussion. However, the risk of publication bias in the effects of current (Figure 2(d)) and the effects of EA combined with other treatments (Figure 2(e)) would affect the quality of their evidence. Except that the risk of publication bias in the comparison of EA versus MA was the same as in the current, the remaining subgroups were unable to draw reliable funnel charts with few studies.

### 3.3. EA versus Other Treatments

**Figure 3:**
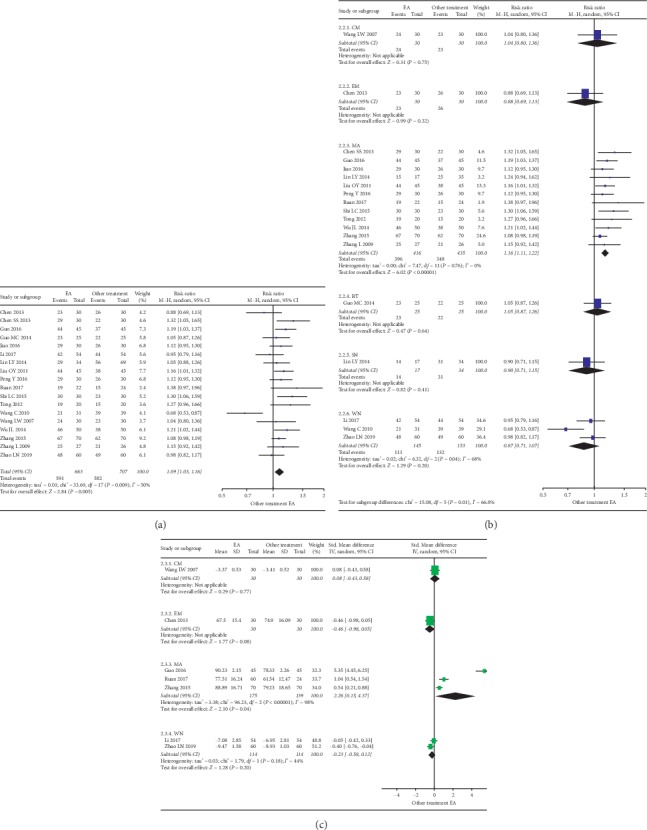
(a) Forest plot of the responding rate difference on EA. (b) Forest plot of the facial nerve function on EA as subgroups by other treatment. (c) Forest plot of the responding rate difference on EA as subgroups by other treatment. *Note*. CM: Chinese massage. EA: electroacupuncture. EM: embedding. MA: manual acupuncture. RT: Rood technique. SN: stuck needling. WN: warm needling.

Eighteen studies reported the results of EA versus other treatments (Figure 3(a)). Generally, EA was significantly more effective in improving the responding rate than control group counterparts (RR: 1.09, 95% CI 1.03 to 1.16; *I*^2^ = 50%; 18 studies, 1370 participants). We performed a sensitivity analysis and found that when 1 study (Wang and Chen [32]) was removed, and the remaining studies were considered homogeneous and the results were stable (RR: 1.11, 95% CI 1.06 to 1.16; *I*^2^ = 12%; 17 studies, 1300 participants). Considering the heterogeneity, we performed subgroup analysis based on the specific treatments used in control groups (Figure 3(b)). Subgroup analysis showed that there were little or no differences in EA versus embedding (RR: 0.88, 95% CI 0.69 to 1.13; 1 study, 60 participants), Chinese massage (RR: 1.04, 95% CI 0.80 to 1.36; 1 study, 60 participants), Rood technique (RR: 1.05, 95% CI 0.87 to 1.26; 1 study, 50 participants), stuck needling (RR: 0.90, 95% CI 0.71 to 1.15; 1 study, 51 participants), or warm needling (RR: 0.87, 95% CI 0.71 to 1.07; *I*^2^ = 68%; 3 studies, 298 participants). EA was significantly more effective in improving the responding rate than MA (RR: 1.16, 95% CI 1.11 to 1.22; *I*^2^ = 0%; 12 studies, 851 participants). The results of the sensitivity analysis of the warm needle subgroup showed that, after removing 1 study (Wang and Chen [32]), the remaining studies could be considered homogeneous, but the stability could not be assessed with only 2 studies (RR: 0.97, 95% CI 0.85 to 1.10; *I*^2^ = 0%; 2 studies, 228 participants).

Seven studies reporting the FNF (scores and EMG results) were analyzed as subgroups (Figure 3(c)). Results showed that EA was not significantly more effective in improving FNF than embedding (SMD: −0.46, 95% CI −0.98 to 0.05; 1 study, 60 participants), Chinese massage (SMD: 0.08, 95% CI −0.43 to 0.58; 1 study, 60 participants), and warm needling (SMD: −0.23, 95% CI −0.58 to 0.12; 2 studies, 228 participants). EA was significantly more effective in improving FNF than MA (SMD: 2.26, 95% CI 0.15 to 4.37; 3 studies, 314 participants).

### 3.4. Current and Frequency

**Figure 4:**
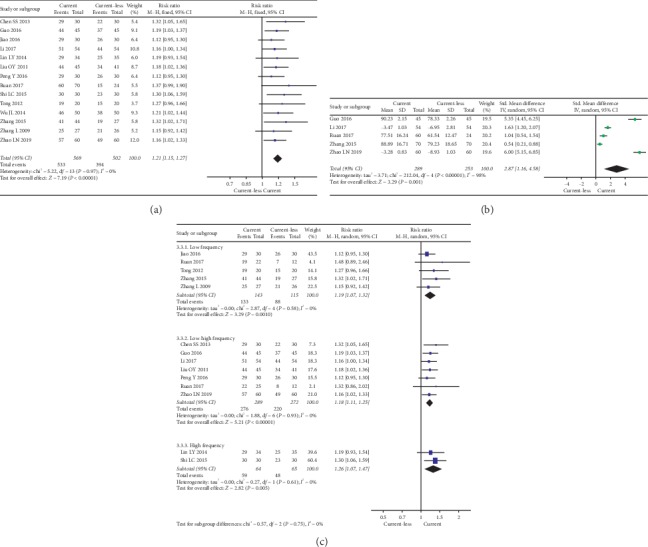
(a) Forest plot of the responding rate difference on current. (b) Forest plot of the facial nerve function on current. (c) Forest plot of the responding rate difference on current characteristics.

Fifteen studies compared EA and current-less acupuncture (e.g., MA and warm needling) were selected to highlight the effect of current (current was the single variable between the experimental and control group). We analyzed the differences in the responding rate (Figure 4(a)) and FNF (Figure 4(b)).

Generally, the presence of current significantly improved the responding rate (RR: 1.21, 95% CI 1.15 to 1.27; *I*^2^ = 0%; 15 studies, 1132 participants). Also, the sensitivity analysis showed that the outcomes were stable. Five studies reported that the current significantly improved the FNF (SMD: 2.87, 95% CI 1.16 to 4.58; 5 studies, 542 participants).

Fourteen studies reported current characteristics (Figure 4(c)). Low frequency (RR: 1.19, 95% CI 1.09 to 1.32; *I*^2^ = 0%; 6 studies, 318 participants), low-high frequency (RR: 1.18, 95% CI 1.11 to 1.25; *I*^2^ = 0%; 7 studies, 561 participants), and high frequency (RR: 1.26, 95% CI 1.07 to 1.47; *I*^2^ = 0%; 2 studies, 129 participants) currents all significantly improved the responding rate. Differences between current frequencies were not significant (*χ*^2^ = 0.58, *P*=0.75; comparing between subgroups). There were no enough data for us to analyze the effect of different current frequencies on FNF.

### 3.5. EA as Part of Combined Therapy versus Controls with No EA

**Figure 5:**
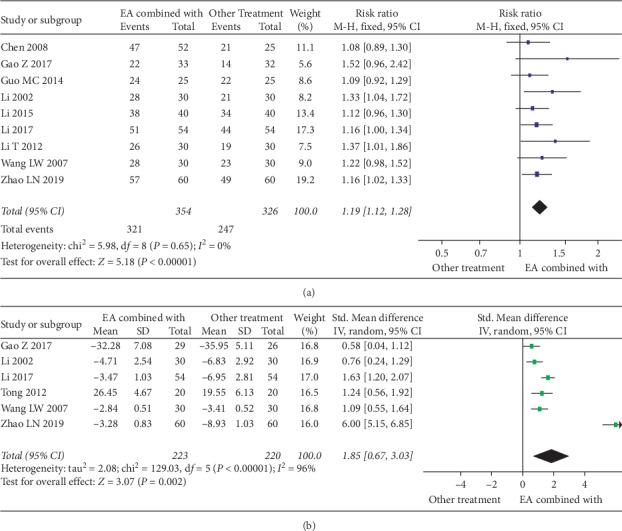
(a) Forest plot of the responding rate differences on EA combined with other treatments. (b) Forest plot of the facial nerve function on EA combined with other treatments.

Nine studies reported the EA combined with other treatments versus control (respective treatments without EA). We analyzed differences in responding rate (Figure 5(a)). Results showed that EA combined therapy was more effective in improving the responding rate (RR: 1.19, 95% CI 1.12 to 1.28; *I*^2^ = 0%; 9 studies, 680 participants). Also, the sensitivity analysis showed that the outcomes were stable. Six of those studies reported the FNF scores (Figure 5(b)) and showed that EA combined therapy was more effective in improving FNF (SMD: 1.85, 95% CI 0.67 to 3.03; 6 studies, 443 participants).

### 3.6. Adverse Events

Only 3 studies [16, 23, 35] reported adverse events. In 1 out of these 3 studies [23], adverse events happened (EA combined with western drugs: 3/40, western drugs: 7/40), with no details presented. There were no enough data for us to analyze the adverse events.

## 4. Discussion

### 4.1. Key Results and Evidence Quality

After analyzing 23 studies covering 1985 participants, our conclusions are as follows: For PFP, EA is superior to MA (low quality); there are no significant differences between EA versus embedding, Chinese massage, Rood technique, stuck needling, or warm needling (very-low quality); low frequency (low quality), low-high frequency (moderate quality), or high-frequency (low quality) electroacupuncture are all effective on PFP; when combined with other treatments, EA effectiveness was improved (low quality).

Across the risk of bias (Figure 2), we determined that the evidence qualities were from very-low to moderate (Table 3). The overall quality is low.

Due to the poor quality of the evidence, the confidence of this meta-analysis is low. However, we have confidence in the following conclusions and believe that their directions will not change; EA is better than MA for PFP; low-high frequency current EA is more effective on PFP, and PFP patients who received EA (combined with other treatments or used separately) could achieve a better prognosis.

### 4.2. Limitations

A variety of causes can lead to bias in acupuncture-related studies [38, 39]. For our analysis, the main limitations come from defects in original studies and regions.

Most studies used random number tables to generate random numbers; though quick and easy, it has potential allocation concealment problems.

Underuse of blinding is another important flaw that cannot be ignored. Sham acupuncture is an alternative to blinding; however, whether sham acupuncture can be used as a real placebo is controversial [40, 41] due to the nature of acupuncture, patients can clearly identify what kind of treatment they are receiving. Although blinding is important to clinical studies, the placebo effect of acupuncture is difficult to analyze separately. Therefore, we believe that the lack of blinding does not seriously impact the curative effect of acupuncture.

The presence of selective reports suggests that some studies might have hidden important negative conclusions. Most, if not all, studies included in this analysis lacked credible “list of observations” which should be found within a protocol or clinical trial registration. Included studies usually put their list of observations in the “Method” section of the research paper, which made us wonder whether these lists were made before or after the actual experiments and unable to rule out the possibility of selective reporting. Some studies [22, 33] had high selective reporting risks (results reported were not mentioned in the method section). Fortunately, selective reporting did not affect our conclusion on primary outcomes; it still had an impact on the overall evidence quality in this meta-analysis.

Studies on acupuncture treatment on facial paralysis are mainly clinical studies from China [42]. All studies and participants involved in this analysis were from China; therefore, we lacked data support from multiple countries and multiple ethnic groups.

## 5. Conclusions and Prospects

Generally speaking, EA is an effective treatment for PFP. Facial acupoints could also be selected for EA therapy to bring benefits to PFP patients. However, we did not find enough evidence to assess the potential harm of EA. We suggest that the use of EA should be fully weighed.

The quality of the evidence found in this meta-analysis is not high, and we suggest more large-sample, rigorous-designed, and standardized clinical trials from different countries and regions be conducted to update our results. Considering the difficulty in blinding the participants and personnel in such trials, future RCTs should work on blinding outcome assessors, more clear random methods, and the inclusion of major patient-important outcomes, such as short-term and long-term effects, sequelae (crocodile tear syndrome, synkinesis, and perversion of facial paralysis), quality of daily life, and adverse events.

## Figures and Tables

**Table 1:** Studied characteristics.

Study	Male/female	Age (years, range)	Age (years, $\bar{x}\pm s$)	The course of disease (days, range)	The course of disease (days, $\bar{x}\pm s$)
Chen [15]	61/60	—	32.63 ± 3.90	—	123.34 ± 12.70
Chen [16]	24/36	18–65	40.06 ± 11.48	78–155	105.97 ± 22.57
Chen et al. [17]	25/35	—	—	<14	—
Gao and Zheng [18]	32/33	7–75	44.73 ± 11.67	<7	2.65 ± 1.45
Guo [19]	42/48	19–68	48.67 ± 4.22	—	—
Guo et al. [20]	37/38	—	40.33 ± 5.75	<90	2.98 ± 0.54
Jiao [21]	27/33	15–70	—	<7	—
Li [22]	15/45	20–60	—	<7	—
Li [23]	51/29	28–63	46.60 ± 0.80	<30	13.85 ± 2.78
Li [24]	84/78	20–64	34.30 ± 7.49	<7	2.35 ± 0.39
Li and Tong [25]	30/30	20–60	—	7–60	—
Lin et al. [26]	50/53	20–60	56.66 ± 19.17	>90	103.15 ± 5.00
Liu et al. [27]	50/36	—	39.48 ± 26.82	—	4.99 ± 3.98
Peng and Yu [28]	35/41	13–78	—	14–47	—
Ruan [29]	35/41	15–70	41.78 ± 12.16	2–17	6.61 ± 3.74
Shi et al. [30]	21/39	20–70	—	21–90	—
Tong [31]	22/18	15–60	34.15 ± 11.51	<30	—
Wang and Chen [32]	34/36	14–68	—	>30	—
Wang et al. [33]	41/49	9–65	—	4–790	—
Wu et al. [34]	38/62	23–63	—	<14	—
Zhang [35]	81/59	0.08–6	2.12 ± 1.43	7–25	8.31 ± 4.95
Zhang and Liu [36]	29/24	8–76	50.47 ± 15.92	<7	4.81 ± 1.76
Zhao et al. [37]	117/63	30–69	43.60 ± 4.40	—	—

*Note*. $\bar{x}\pm s$: mean and standard deviation, —: no data.

**Table 2:** Design of studies.

Study	References	Code	Study type	Participants	Experimental group	Characteristic of current	Control group	Arms
Chen	[15]	Chen 2008	RCT	121	EA/IT/CM	L	IT/CM	4
Chen	[16]	Chen 2013	RCT	60	EA	LH	EM	2
Chen et al.	[17]	Chen SS 2013	RCT	60	EA	LH	MA	2
Gao and Zheng	[18]	Gao Z 2017	RCT	65	EA/SD	L	SD	2
Guo et al.	[19]	Guo 2016	RCT	90	EA	LH	MA	2
Guo	[20]	Guo MC 2014	RCT	75	EA/RT	LH	RT	3
Jiao	[21]	Jiao 2016	RCT	60	EA	L	MA	2
Li	[22]	Li 2002	RCT	60	EA/SD	LH	SD	2
Li	[23]	Li 2015	RCT	80	EA/SD	LH	SD	2
Li	[24]	Li 2017	RCT	162	EA/WN	LH	WN	3
Li and Tong	[25]	Li T 2012	RCT	60	EA/SD	L	SD	2
Lin et al.	[26]	Lin LY 2014	RCT	103	EA	H	MA/SN	3
Liu et al.	[27]	Liu OY 2011	RCT	86	EA/TCMD	LH	MA/TCMD	2
Peng and Yu	[28]	Peng Y 2016	RCT	76	EA	LH	MA	2
Ruan	[29]	Ruan 2017	RCT	94	EA/IT	L/LH/DC	MA/IT	4
Shi et al.	[30]	Shi LC 2015	RCT	60	EA	H	MA	2
Tong	[31]	Tong 2012	RCT	40	EA/SD	L	MA/SD	2
Wang and Chen	[32]	Wang C 2010	RCT	70	EA	LH	WN	2
Wang et al.	[33]	Wang LW 2007	RCT	90	EA/CM	L	CM	3
Wu et al.	[34]	Wu JL 2014	RCT	100	EA	-	MA	2
Zhang	[35]	Zhang 2015	RCT	140	EA	L	MA	2
Zhang and Liu	[36]	Zhang L 2009	RCT	53	EA	L	MA	2
Zhao et al.	[37]	Zhao LN 2019	RCT	180	EA/WN	LH	WN	3

**Table 3:** Evidence quality of the results of responding rate.

Results	Test type	Downgrade factor					Upgrade factor			Quality
		DD	In	HE	RI	PB	SE	BSE	DE	
*EA versus “controls”*										
Total	RCT	−1^A^	0	−1^B^	0	0	0	0	0	Low
Embedding	RCT	−1^A^	0	0	−2^C^	0	0	0	0	Very low
Massage	RCT	−1^A^	0	0	−2^C^	0	0	0	0	Very low
Needle acupuncture	RCT	−1^A^	0	0	0	0	0	0	0	Moderate
Rood technique	RCT	−1^A^	0	0	−2^C^	0	0	0	0	Very low
Stuck needling	RCT	−1^A^	0	0	−2^C^	0	0	0	0	Very low
Warm needling	RCT	−1^A^	0	−1^B^	−1^D^	0	0	0	0	Very low
Current	RCT	−1^A^	0	0	0	−1^F^	0	0	0	Low
*Current characteristics*										
Low frequency	RCT	−1^A^	0	0	−1^D^	0	0	0	0	Low
Low-high frequency	RCT	−1^A^	0	0	0	0	0	0	0	Moderate
High frequency	RCT	−1^A^	0	0	−1^D^	0	0	0	0	Low
DBC	RCT	−1^A^	−1^E^	0	0	0	0	0	0	Low
EA as an adjunct	RCT	−1^A^	0	0	0	−1^F^	0	0	0	Low

*Note*. DD: design defects. IN: indirectness. HE: heterogeneity. RI: data sparse or incomplete. PB: publication bias. SE: significant effect. BSE: bias subtractive effect. DE: dose effect. DBC: differences between current characteristics. A: study design defects may affect the results; B: cannot ignore heterogeneity; C: very few participants seriously affect the results; D: very few participants may affect the results; E: indirect comparison results; F: publication bias may affect the results.

## Data Availability

Most of the data information is fully reflected in the article (paritcipants, age, gender, scale scores, risk of bias, etc.). As a supplement, we uploaded a RevMan file (.rm5) containing the original data.

## Supplementary Materials

Supplementary Materials The checklist is based on the PRISMA-checklist provided by EQUATOR. Codebook is a screening (encoded) of the original studies by our author team.
